# No self-advantage in recognizing photographs of one’s own hand: experimental and meta-analytic evidence

**DOI:** 10.1007/s00221-022-06385-9

**Published:** 2022-05-20

**Authors:** Nicholas P. Holmes, Charles Spence, Yves Rossetti

**Affiliations:** 1 School of Psychology, University of Nottingham, Nottingham, NG7 2RD UK; 2 Department of Experimental Psychology, University of Oxford, Oxford, UK; 3 Equipe ‘Trajectoires’, Centre de Recherche en Neurosciences de Lyon, Inserm UMR-S 1028, CNRS UMR 5292, Université de Lyon, Université Lyon 1, Bron, France; 4 Plate-Forme ‘Mouvement et Handicap’, hôpital Henry Gabrielle, Hospices Civils de Lyon, Saint-Genis-Laval, France; 5 Service de médecine physique et réadaptation, hôpital Henry Gabrielle, Hospices Civils de Lyon, Saint-Genis Laval, France

**Keywords:** Self-recognition, Hand perception, Reaction time, Meta-analysis

## Abstract

Visually recognising one’s own body is important both for controlling movement and for one’s sense of self. Twenty previous studies asked healthy adults to make rapid recognition judgements about photographs of their own and other peoples’ hands. Some of these judgements involved explicit self-recognition: “Is this your hand or another person’s?” while others assessed self-recognition implicitly, comparing performance for self and other hands in tasks unrelated to self-other discrimination (e.g., left-versus-right; match-to-sample). We report five experiments with three groups of participants performing left-versus-right (Experiment 1) and self-versus-other discrimination tasks (Experiments 2 to 5). No evidence was found for better performance with self than with other stimuli, but some evidence was found for a self-disadvantage in the explicit task. Manipulating stimulus duration as a proxy for task difficulty revealed strong response biases in the explicit self-recognition task. Rather than discriminating between self and other stimuli, participants seem to treat self-other discrimination tasks as self-detection tasks, raising their criterion and consistently responding ‘not me’ when the task is difficult. A meta-analysis of 21 studies revealed no overall self-advantage, and suggested a publication bias for reports showing self-advantages in implicit tasks. Although this may appear counter-intuitive, we suggest that there may be no self-advantage in hand recognition.

**Supplementary Information:**

The online version contains supplementary material available at 10.1007/s00221-022-06385-9.

## Introduction

The English expression “I know it like the back of my hand” suggests that we are familiar with the appearance of our own hands and are able to recognise them. Studies of human self-recognition have focussed mainly on faces (e.g., Keenan et al. 2000), but there are at least 67 studies of hand and other body-part recognition in healthy adults. The earliest study may be that of Nielsen (1963), who tricked his participants into believing they were viewing their own hand drawing, when, sometimes, it was in fact the experimenter’s hand. Nielsen observed participants making large errors as the experimenter’s hand moved off course, but participants also confabulated about why they had erred, failing to detect the experimenter’s hand or the visual-proprioceptive mismatch. Using looking behaviour, Bahrick and Watson (1985) suggested that infants discriminate self from other using visual-proprioceptive congruency. Many later studies have investigated self-monitoring, self-recognition and agency by manipulating the mismatch between self-generated movements and their visual consequences, or even the mismatch between hand movements and the explicit detection of these movements (Pélisson et al. 1986; Fourneret and Jeannerod 1998; see the supplementary spreadsheet for a full list).

A smaller literature has asked how well participants can recognise photographs of their own hands. Wuillemin and Richardson (1982) showed participants an array of objects including a secretly taken picture of one of their own hands. Most participants did not pick their own hand when asked to select familiar items, although they were able to distinguish them from others when asked explicitly. Most relevant to the current work, a series of 20 studies, beginning with Frassinetti et al. (2008) presented participants with colour (Aranda et al. 2010; Conson et al. 2010; Fukui et al. 2020; Kuroki and Fukui 2020; Sanabria et al. 2015; Salerno et al. 2012; Su et al. 2010) or greyscale (Campione et al. 2017; Candini et al. 2016; Conson et al. 2015, 2017; De Bellis et al. 2017; Ferri et al. 2011; Galigani et al. 2021; Malaspina et al. 2019; Olgiati et al. 2017) images of hands (or hands and other body parts, Frassinetti et al. 2009, 2010, 2011, 2012) and asked them to make either explicit self-recognition judgements (self vs. other), or to make left versus right, same versus different, or match-to-sample judgements. In these ‘implicit’ tasks, researchers have often reported a ‘self-advantage’ effect, whereby participants perform the task faster and/or with fewer errors when responding to images of their own than other people’s hands. Following a systematic review of the literature, we found 17 reports of differences between self and other stimuli in reaction times (RT) and percentage of errors for implicit self-recognition tasks, and 9 for explicit tasks. For implicit tasks, the differences ranged from a self-advantage of 133 ms to a self-disadvantage of 4 ms, while errors ranged from an 11% advantage to a 2% disadvantage. Explicit tasks were less consistent, ranging from a 61 ms and 16% advantage to a 270 ms and 15% disadvantage.

The present study was originally designed (in 2005) to compare the hand self-recognition ability of healthy adults to that of brain-damaged participants, making both implicit (left-versus-right) and explicit (self-versus-other) hand recognition judgements. Since insufficient and highly variable data were collected from the neurological group, the overall study was not written-up in detail. There is, however, now a substantial literature on hand self-recognition in healthy participants, so the data from healthy participants have been re-analysed and accompanied by a systematic review and meta-analysis. The present study asks, experimentally and meta-analytically: is there a self-advantage in the recognition of hand images in implicit and explicit tasks? Following Keenan et al.’s (2000) hemispheric dominance hypothesis, we also designed experiments to ask whether performance is better when our left hand responds to self and right hand to other images, as compared to the opposite mapping. Finally, we assessed the influence of task difficulty (operationalised in terms of stimulus duration), and the effect of informing participants about the proportions of self-images that would be presented. Both of these manipulations were designed to assess the presence of response biases in hand self-recognition.

## Methods

### Participants

Three groups of healthy adult participants completed a range of tasks across five experiments. Participants were recruited in 2005–2006 from the local population of staff and students in Lyon; all participants were (likely) white/Caucasian Western Europeans, although this information was not requested or recorded. Handedness was assessed with the 20-item version of the Edinburgh Inventory (Oldfield 1971), but no additional data were collected about how often participants worked or how skilled they may be with their hands. Details of participants and experimental design for all experiments are given in Table 1. All participants gave their written informed consent. Some were financially compensated for their time, others participated without compensation. The experiments were conducted under local ethical approval which, at the time, did not require a formal study-specific assessment. Rather, the experimenters and supervisors assessed the ethical implications of the work and proceeded under a “blanket” approval. No prospective power analysis was done; the sample size for Experiments 1 and 2 was determined by convenience.

**Table 1:** Experimental design and participants

E	*N*	*F*	Age (y) Mean (SD)	LQ mean (SD)	Task	Responses	Stimulus duration (ms)	Mask duration (ms)	P(self)	Trials
1a	15*	9	28.2 (4.74)	73.9 (26.7)	LR	Index and middle fingers	1000	0	0.33	120
1b							17–533	200		288
2a					SO		1000	0	0.33	120
2b							17–533	200		288
3	7	4	24.7 (3.31)	63.2 (57.4)	SO	Left and right index fingers	17–533	200	0.50	384
4	28	19	29.4 (5.61)	75.8 (34.6)	SO		1000	0	0.50	128
5a							17–533	200	0.25	96
5b									0.50	96
5c									0.75	96

*E* experiment, *N* number of participants, *F* number of females, *y* years, *LQ* laterality quotient, *LR* left versus right, *SO* Self versus Other, *P(Self)* proportion of self-stimuli

*One participant did not complete the second task, so an additional participant was recruited, leaving *N* = 14 per task

### Apparatus and materials

Stimuli were presented on a laptop computer (Toshiba Tecra, monitor = 1024 × 768 pixels, 285 × 215 mm, 60 Hz), running Presentation 0.81 (Neurobehavioural Systems, Albany, USA). Responses were collected with a high-speed USB-2.0 mouse.

Four flash photographs of each participant’s hands (left and right, in palm-down and palm-up postures) were taken with an Olympus FE-100 digital camera, from vertically above, close to a natural light source. The photos were digitally edited (Corel Photopaint), including: creating a black background; centring the hand in a 420(*w*) × 510(*h*) pixel frame (13.5 × 17.5 cm, ~ 11.8 × 15.4° on screen); equalising hand length across participants (350 pixels from the wrist to the middle fingertip), and digitally removing a ring in one participant, trimming long fingernails in 16 participants, and editing-out 2 red areas of skin (due to a burn or swelling) on two participants’ hands. Images were saved as 72dpi 24-bit RGB JPEG files. A mask image montage of four different scrambled hand images was also created.

The luminance, contrast, and RGB colour channels of each image were adjusted by the experimenter (NPH) to minimise stimulus differences amongst the hand images, separately for each participant.

### Design

Experiments 1–3 followed a within-participants, repeated measures design, while Experiments 4 and 5 also contained between-participants variables. Each block comprised trials defined by the factorisation of the repeated measures variables: Task (Experiment 1: left vs. right; Experiments 2–5: self vs. other), stimulus hand (left, right), stimulus posture (palm-down, palm-up), and stimulus identity (self, other1, other2). The stimuli consisted of photos of each participant’s and two other participants’ hands, matched to the participant’s gender. The other hands were chosen by the experimenter to be visually as similar as possible in shape, size, skin colour and posture as the participant’s own hands (i.e., to make the discrimination task as difficult as possible). Where possible, the other hands were taken from participants in the same experiment, however, priority was given to choosing hands as similar in appearance as possible to the participant’s hands.

**Fig. 1:**
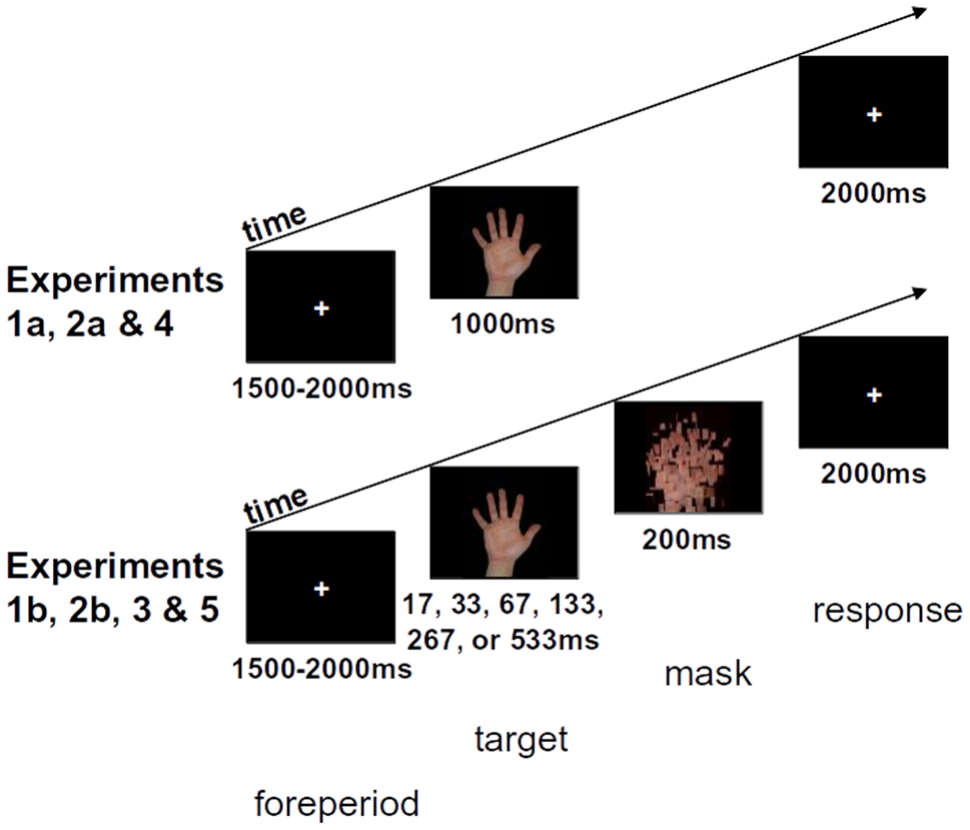
Experimental design. A randomised interval of 1500–2000 ms preceded each trial. One of 12 hand images (left or right hand, palm-down or palm-up posture, belonging to the participant or to one of two other participants) was then presented. The participant had to respond within 2 s with a button-press according to whether the hand was a left or right hand (Experiments 1a and 1b), or whether the hand was their own or another person’s (Experiments 2–5). In Experiments 1b, 2b, 4 and 5, stimulus duration was varied from 17 to 533 ms (1 to 32 screen refreshes), and a 200 ms mask followed the stimulus. In Experiments 1a, 2a and 4, stimuli were displayed for 1000 ms, and no mask was presented

There were 12 main stimulus conditions. For analysis, data from the two ‘other’ conditions were pooled, giving eight conditions. In Experiments 1a, 2a and 4, stimuli were presented for 1000 ms, followed by a fixation cross, in pseudorandom order, five times per block for two blocks (total = 120 trials). In Experiments 1b, 2b, 3 and 5, stimuli were presented for one of six durations (17, 33, 67, 133, 267 or 533 ms, on a 60 Hz screen), followed by a mask (Fig. 1), in pseudorandom order twice per stimulus duration per block for four blocks (total = 288 trials).

In Experiments 1 and 2, the participants responded with the index and middle fingers of the left or right hand, while in Experiments 3, 4 and 5, both left and right index fingers were used. All stimulus–response mappings and hands were counterbalanced. Experiments 1 and 2 were conducted separately, in two counterbalanced sessions a mean ± SD of 6.23 ± 11.0 days (range 1–33 days) apart. One participant did not return for the second experiment, so an additional participant was recruited. Experiments 3, 4 and 5 took place separately.

### Procedure

Participants sat in a quiet, dimly-lit room with their eyes ~ 50 cm from the centre of the screen, holding a computer mouse underneath a platform which hid their hands from view. In Experiments 1 and 2, the participants held their responding forearm parallel and response buttons arranged perpendicular to the screen to minimise spatial compatibilities between the stimuli and responses. In Experiments 3, 4 and 5, left and right hands were orthogonal to the screen, one index finger on each mouse button.

Each trial began with a white central fixation cross (1.2 × 1.2 cm, ~ 1 × 1º) for a pseudorandom 1500–2000 ms before the target. One of the 12 hand stimuli was presented centrally for 1000 ms (Experiments 1a, 2a and 4) or between 17 and 533 ms (Experiments 1b, 2b, 3 and 5). The participants were given 2000 ms to respond according to whether the presented hand was a left or a right hand (Experiment 1) or their own or another person’s hand (Experiments 2–5). Stimuli were followed by a fixation cross (Experiments 1a, 2a and 4) or a 200 ms masking stimulus then a fixation cross (Experiments 1b, 2b, 3 and 5). The participants were instructed to respond as rapidly as possible, and to minimise errors, by pressing one of the two buttons according to the task instructions.

### Analysis

The mean correct reaction times (RT) and the proportion of errors were recorded for each condition. RT distributions were positively skewed, so raw RT data were log10-transformed before averaging. Mean RTs were transformed back before statistical analysis. A small number of extreme datapoints were removed—in all cases, these were due to technical failures or missed responses (e.g., RT < 100 ms, RT ≥ 2000 ms).

The main question was whether performance was better for self than other hands, for which data were averaged over all other conditions, and paired t tests were used. The self-advantage effect should result in better performance (shorter RTs, fewer errors) for self than other hands, particularly in implicit (left vs. right) tasks. For Experiments 1b, 2b, 3 and 5, data from each participant were fit with log-transformed (RT) or logistic (error) regression. Pooled across all conditions and participants, fits were marginally better for RT on a log compared to a linear scale, and logistic fits were substantially better than linear fits for error data, so these were used throughout. The fit parameters (intercept, slope, and Z-transformed r values) were compared between self and other conditions to assess how stimulus duration (i.e., task difficulty) affected participants’ responses.

### Experiments

Experiment 1 required participants to perform left-versus-right discriminations with 1000 ms unmasked stimuli (E1a) and variable duration masked stimuli (E1b). Experiment 2 was the same, but required self-versus-other discriminations instead. The purpose of Experiments 1 and 2 was to examine performance for self and other stimuli in implicit and explicit discrimination tasks, and under a difficulty manipulation. Stimulus presentation duration was manipulated as a proxy for task difficulty, to test if participants systematically changed how they responded to self-other and left–right tasks. If there are no response biases, then performance in all conditions should be around 50% when the task is very difficult. In contrast, if participants show systematic response biases, performance will deviate significantly from 50% when the task is difficult. Experiments 1 and 2 were run on the same participants in counterbalanced order.

In Experiments 1 and 2, one-third of the target stimuli were self-images and two thirds others. The participants were not informed of these proportions, but if they had discovered the proportions, they might have used this information to adjust how they guessed when the task was difficult. This might have led to ~ 67% other and 33% self-responses for the shortest durations. To test this, Experiment 3 was a short replication of Experiment 1 in a subset of those participants, with equal proportions of ‘self’ and ‘other’ stimuli. The stimulus–response mapping across hands was also counterbalanced (self-left/other-right vs. self-right/other-left). Data collection in Experiment 3 was terminated early, once it became clear that the pattern of results was unchanged. Instead, we designed a fourth and fifth experiment to test if participants could change their response criterion in accordance with the likely proportions of self and other stimuli.

Experiments 4 and 5 were run on the same group of 28 participants, with Experiment 4 always first. Eight had taken part in Experiments 1 and 2, and six in Experiments 1, 2 and 3. Experiment 4 tested for any systematic association between self and other stimuli and left and right responses. Returning participants were assigned to the same stimulus–response mapping as they received in Experiments 1, 2 and/or 3. One possibility for a self-left and right-other advantage could be a right hemisphere dominance for self-processing, assuming that images of left hands are processed more by the right hemisphere, even when presented centrally (Keenan et al. 2000). Another possibility is an implicit association or compatibility between self and left and/or between other and right hands. Potential associations include word length and letter overlap in English (s-E-L-F and L-E-F-t; o-T-H-e-R and R-i-g-H-T), the order in which these words are typically used: self-other, left–right, and the positions of ‘S’ and ‘O’ on keyboards (but not ‘L’ and ‘R’!). These task-irrelevant associations may generate RT or accuracy benefits for particular combinations of stimuli and responses. However, the experiments were carried out with mostly French- and Italian-speaking participants instructed, mostly, in English. Experiments 4 and 5 explicitly tested the self-left/other-right against the self-right/other-left mapping.

Experiment 5 tested the influence of prior knowledge about the likelihood of self and other images. The strong biases observed in Experiments 2–4 to respond ‘other’ when the task was difficult, could have been due to participants’ assumptions about the likely number of ‘self’ stimuli. Half of the participants (‘Naive’) were told only that the three blocks of trials contained their own and other people’s hands, and that the three blocks were different in some way. The other half (‘Informed’) were told the percentage of self-stimuli that would appear in each block, and were encouraged to use this information to improve. Three blocks of trials with 25%, 50%, and 75% self-stimuli were run in counterbalanced order. The aim of Experiment 5 was to assess if different proportions of self and other stimuli would affect participants’ likelihood of responding ‘self’, and whether explicit instruction about these proportions affects their responses.

### Meta-analysis

To compare current and published results, PubMed was searched with the query: “(visual* OR vision OR imag*) AND (hand AND (recogni* OR identi*)) AND (self OR other) AND (behaviour and behaviour mechanisms[MeSH Terms])”. On 31st March 2021, this returned 1135 results, and when repeated on 3rd January 2022, 1197. The results were filtered first by screening titles (283 included), then abstracts (56). Two articles could not be retrieved, so 54 full texts were checked (22 included). 51 articles were added based on prior research and checking reference lists. 6 duplicates were removed, leaving 67 articles with a total of 100 experimental groups.

The 67 studies were read and the experimental manipulations classified according to whether self and other hand images (20 included) or videos (22 excluded), virtual hands (10 excluded), or cursors (11 excluded) were used. Four further studies were excluded, because no self- versus other analysis was possible. Of the 20 included studies (33 experimental groups), 7 involved full colour photos and 13 greyscale. Of the 13 greyscale studies, 4 included a mix of non-face body parts (e.g., hands, legs). Within these mixed studies, the authors reported no between-body-part differences, so the data were pooled with all other exclusively hand studies. The full list of 1135 articles screened, along with a classification and more extensive notes for all 67 potentially relevant reports is provided in the supplementary spreadsheet file (meta_analysis.ods), specifically in the ‘DETAILS’ tab. A PRISMA flowchart is given in the supplementary materials.

Effect sizes for self-versus-other comparisons were calculated from the included studies, using information from figures and text, and converting statistical results where necessary. This yielded 27 effect sizes (13 with SE) for percentage error, and 26 for RT (17 with SE). Effect-sizes from the current studies were added. Random-effects meta-analysis in JASP 0.9.2 was conducted separately for RT and error data, for all studies together, and for explicit and implicit tasks separately (6 analyses, Bonferroni-corrected alpha = 0.0083). They were also repeated after excluding data from the present report. Overall effect-size, funnel plot regression (sei), and effect-size after trim-and-fill were interpreted to assess potential publication biases.

## Results

### No differences between self and other for implicit or explicit discrimination

**Fig. 2:**
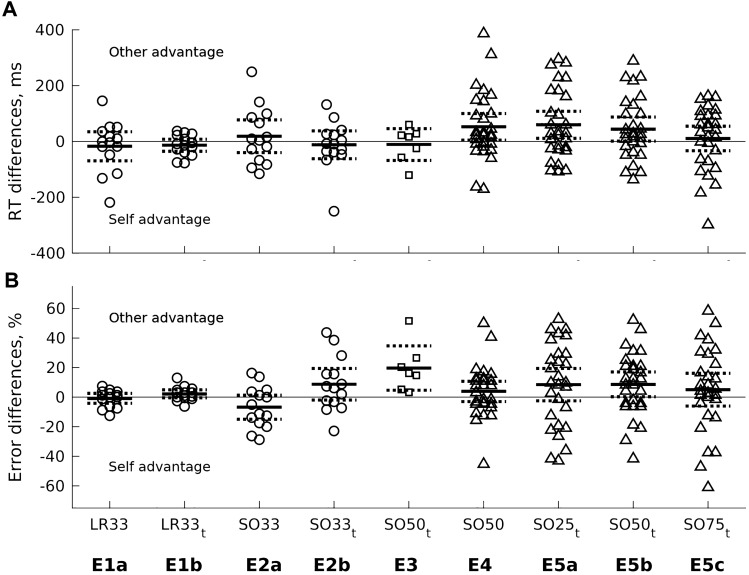
No evidence for a self-advantage across three experimental groups. Data show individual differences between self and other stimuli across a range of experimental conditions for left-versus-right (LR) and self-versus-other (SO) discrimination tasks. Numbers in condition labels refer to the proportion of ‘self’ stimuli (e.g., LR33 = left-versus-right task with 33% self, 67% other stimuli). None of the experimental conditions (E1a to E5c) showed a significant self-advantage effect in either RT (**A**) or percentage errors (**B**). Some conditions showed a significant self-disadvantage in RTs (SO50, SO25_t_, SO50_t_) or errors (SO50, SO50_t_), Table 2. Subscripted t: stimuli were presented for varying durations. Solid horizontal lines: Means; broken horizontal lines: 95% confidence intervals (showing the two-tailed t-test comparison with zero). Negative values on the y-axes show a ‘self-advantage’ and positive a ‘self-disadvantage’. Circles: Group 1; Squares: Group 2; Triangles: Group 3. *ms* milliseconds

Across the three experimental groups and nine conditions, there were no significant self-advantages in either implicit or explicit hand discrimination tasks (Fig. 2). There were some significant self-disadvantages, in Experiment 3 (mean ± SE error difference = 19.7 ± 6.13, *t*(6) = 3.21, *p* = 0.018), Experiment 4 (RT = 52.4 ± 23.2 ms, *t*(27) = 2.26, *p* = 0.032), and three significant differences in Experiment 5 (Table 2), however, none of these differences was very strong.

**Table 2:** Self versus other performance

E	Task	P(Self)	Dur (ms)	Self		Other	
				RT ms (SE)	Error% (SE)	RT ms (SE)	Error% SE
1a	LR	0.33	1000	904 (59.7)	9.29 (1.32)	921 (69.5)	10.1 (1.33)
1b		0.33	17–533	823 (42.6)	22.0 (1.85)	836 (45.7)	19.7 (1.47)
2a	SO	0.33	1000	774 (51.3)	12.1 (2.93)	756 (40.4)	18.9 (3.4)
2b		0.33	17–533	697 (37.3)	32.7 (3.86)	709 (35.9)	23.9 (3.07)
3	SO	0.50	17–533	663 (46.2)	37.8 (4.65)	674 (57.2)	18.1 (1.92)
4	SO	0.50	1000	886 (47.3)	18.5 (2.29)	834 (48.2)	14.6 (2.16)
5a		0.25	17–533	818 (54.1)	41.2 (3.33)	759 (43.1)	32.7 (2.89)
5b		0.50		801 (49.3)	43.6 (2.55)	757 (42.5)	35.0 (2.85)
5c		0.75		771 (45.3)	41.7 (3.11)	760 (42.5)	36.6 (3.84)

E	Self-other difference						
	*N*	RT ms (SE)	*t*	*p*	Error % (SE)	*t*	*p*
1a	14	− 17.5 (24.1)	− 0.73	0.481	− 0.804 (1.55)	− 0.52	0.614
1b		− 13.5 (10.0)	− 1.35	0.200	2.24 (1.25)	1.79	0.097
2a		18.8 (27.1)	0.69	0.501	− 6.79 (3.76)	− 1.81	0.094
2b		− 12.1 (23.3)	− 0.52	0.610	8.72 (4.95)	1.76	0.101
3	7	− 11.0 (23.3)	− 0.47	0.654	19.7 (6.13)	3.21	0.018
4	28	52.4 (23.2)	2.26	0.032	3.91	1.17	0.253
5a		59.8 (23.5)	2.54	0.017	8.48 (5.33)	1.59	0.123
5b		44.1 (20.9)	2.11	0.045	8.63 (4.09)	2.11	0.044
5c		10.3 (21.4)	0.48	0.636	5.06 (5.41)	0.94	0.99

*E* experiment and condition, *P(Self)* proportion of self-stimuli, *Dur.* stimulus duration, *RT* reaction time, *ms* milliseconds, *t* t-statistic for Self-Other Difference; Negative RT, error and *t*-statistics = self-advantage; *p*: *p* value for Self-Other difference. Data are means (SE)

### Task difficulty affects explicit and implicit discrimination differently

**Fig. 3:**
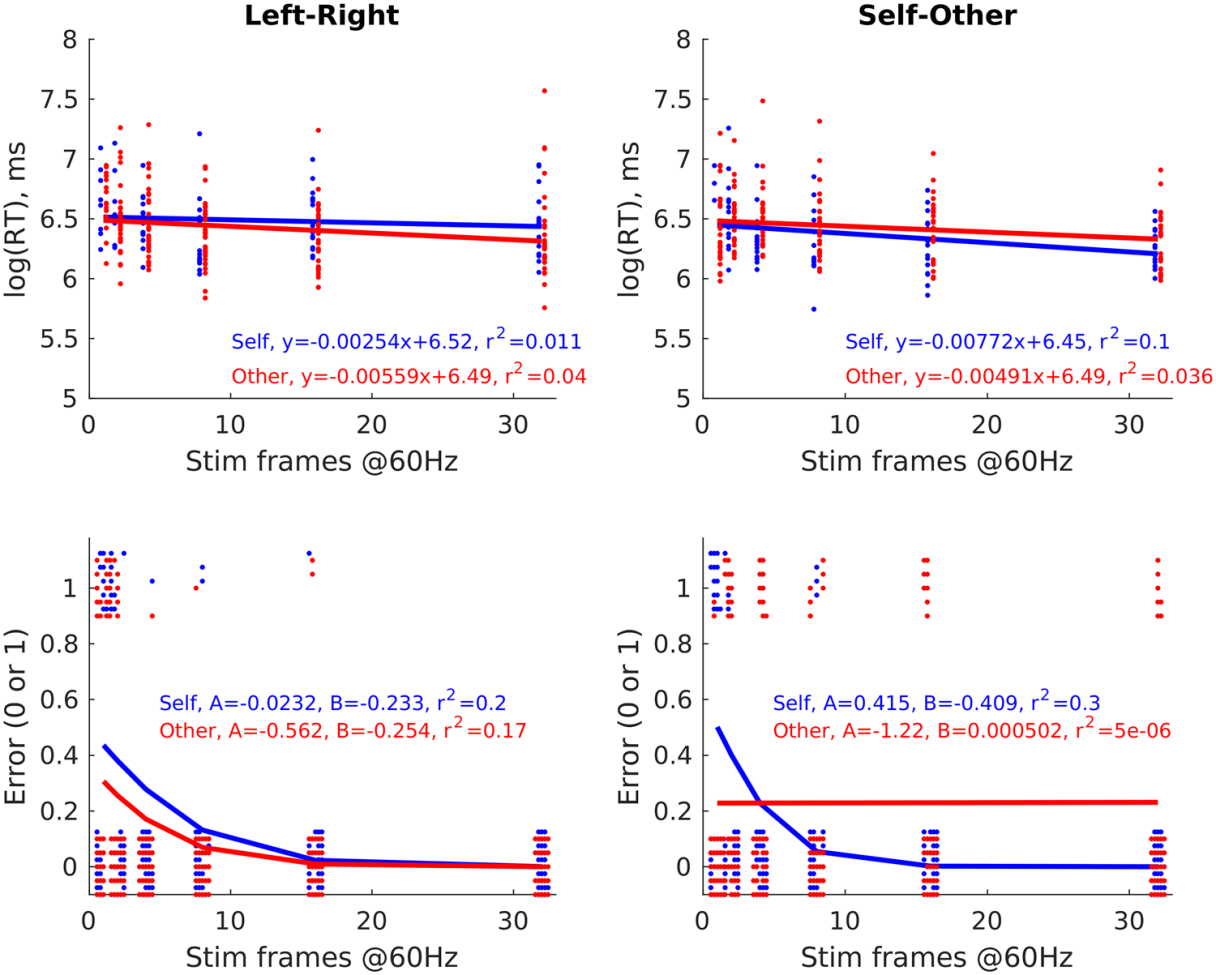
Example participant reaction time (RTs) and error fits as a function of stimulus duration (i.e., task difficulty). Panels **A** and **B** show log10-transformed RT data, **C** and **D** show error data. The left column shows data for the left versus right task (Experiment 1b), the right column for the self-versus other task (Experiment 2b). Linear and logistic fits and equations are shown for self (blue) and other (data) separately. Stim frames @60 Hz—how long the stimulus was presented for, in video frames. This *x*-axis and analysis method compensated for occasional computer errors in stimulus duration. Each datapoint represents one response. Datapoints have been jittered in *x* (RT, error) and *y* (error) axes for display only. Model fits were performed with non-jittered data

In Experiments 1b, 2b, 3 and 5, log10(RT) and error data were fit with linear and logistic regressions, respectively, against target duration. An example of the fitting for one representative participant is shown in Fig. 3. From each fit, three parameters were extracted: intercept, slope and Pearson’s *r* value (converted to a *Z*-score to allow parametric analysis). These parameters were compared statistically between self and other conditions.

In Experiments 1b and 2b, there were no significant differences in fit parameters between self and other conditions for log10(RT) data (Table 3). For error data, intercepts were consistently higher, slopes more negative, and *Z*-transformed *r* values more negative for self than for other stimuli; this was significant for all three fit parameters in the self-versus other, and for the intercept parameter only in the left-versus-right task (Table 4). These patterns are seen in the single participant’s data in Fig. 3—a stronger effect of stimulus duration on errors for self than for other stimuli. This self-bias was stronger in the self-versus-other task than the left-versus-right task, for errors (intercepts, *t*(12) = 3.14, *p* = 0.009; slopes, *t*(12) = 1.76, *p* = 0.104; *Z*-scores, *t*(12) = 3.34, *p* = 0.006), but much less strongly and not significant for log10(RT) (intercepts, *t*(12) = 0.31, *p* = 0.762; slopes, *t*(12) = 0.74, *p* = 0.473; *Z*-scores, *t*(12) = 0.55, *p* = 0.954).

**Table 3:** Self–other differences in regression fit parameters for reaction times

Condition	*N*	Intercepts			Slopes			*Z*-scores		
		*M*	*t*	*p*	*M*	*t*	*p*	*M*	*t*	*p*
LR33	14	− 0.001 (0.013)	− 0.04	0.97	− 0.002 (0.002)	− 1.01	0.332	− 0.034 (0.036)	− 0.95	0.358
SO33		0.010 (0.027)	0.372	0.716	− 0.003 (0.002)	− 1.48	0.162	− 0.079 (0.051)	− 1.54	0.148
SO50	7	0.013 (0.028)	0.47	0.655	− 0.001 (0.002)	− 0.69	0.518	− 0.101 (0.081)	− 1.24	0.262
SO25	28	0.091 (0.035)	2.57	0.016	− 0.003 (0.002)	− 2.16	0.040	− 0.144 (0.062)	− 2.32	0.028
SO50		0.071 (0.030)	2.34	0.027	− 0.002 (0.001)	− 1.67	0.107	− 0.095 (0.059)	− 1.61	0.119
SO75		0.055 (0.033)	1.69	0.102	− 0.005* (0.002)	− 0.90	0.379	− 0.023* (0.068)	− 0.33	0.741
SO Naive	14	0.024 (0.033)	0.75	0.469	− 0.001 (0.001)	− 1.21	0.247	− 0.047 (0.055)	− 0.85	0.409
SO Informed	14	0.120 (0.032)	3.72	0.003	− 0.005 (0.002)	− 2.08	0.058	− 0.200 (0.098)	− 2.04	0.062

N: number of participants. M: Mean Self-Other difference. t: t test statistic. p: p value. LR33: Left–right discrimination task with 33% self stimuli. SO: Self-other discrimination task. Data are means(SE). *One extreme outlying fit parameter was removed, without affecting the conclusion of no difference. The data are plotted in Supplementary Fig. 1

**Table 4:** Self–other differences in regression fit parameters for percentage errors

Condition	*N*	Intercepts			Slopes			*Z*-scores		
		*M*	*t*	*p*	*M*	*t*	*p*	*M*	*t*	*p*
LR33	14	29.0 (11.1)	2.61	0.022	− 3.51 (2.53)	− 1.39	0.188	− 0.039 (0.021)	− 1.88	0.082
SO33		158 (45.2)	3.50	0.004	− 17.8 (5.59)	− 3.18	0.007	− 0.367 (0.096)	− 3.82	0.002
SO50	7	183 (57.3)	3.19	0.019	− 8.64 (5.90)	− 1.46	0.194	− 0.371 (0.147)	− 2.53	0.045
SO25	28	184** (45.4)	4.06	< 0.001	− 22.0 (5.82)	− 3.78	< 0.001	− 0.634 (0.114)	− 5.56	< 0.001
SO50		93.5 (32.9)	2.84	0.008	− 3.86 (3.84)	− 1.01	0.323	− 0.278 (0.103)	− 2.71	0.012
SO75		− 4.34* (48.5)	− 0.09	0.929	8.63 (4.94)	1.75	0.092	0.079 (0.124)	0.64	0.531
SO Naive	14	46.0* (45.2)	1.02	0.331	− 3 (123)	− 0.02	0.981	− 23.6 (12.1)	− 1.95	0.073
SO Informed	14	145^ (51.8)	2.80	0.016	− 51.6 (43.6)	− 1.19	0.257	− 32.0 (15.2)	− 2.11	0.055

N: number of participants. M: Mean Self-Other difference. t: *t* test statistic. p: p value. LR33: Left–right discrimination task with 33% self-stimuli. SO: Self-other discrimination task. *One extreme outlying fit parameter was removed, without affecting the conclusion of no difference. ^One extreme outlying fit parameter was removed, changing the statistics from M ± SE = 315 ± 177 (*t*(13) = 1.78, *p* = 0.098. **Two extreme outlying fit parameters were removed (*Z* = 3.29,3.75). The difference statistics changed from M ± SE = 703 ± 363 (*t*(27) = 1.94, *p* = 0.063. The data are plotted in Supplementary Fig. 2

This bias was replicated in Experiments 3 and 5—as target duration decreased, participants increased their likelihood of responding ‘other’, making more errors for self, and fewer for other stimuli. Significant differences between self and other were found for 5 out of 18 RT and 8 out of 18 error measures, all in the same direction. A signal detection analysis of d-prime, criterion, and ROC curves is provided in supplementary materials.

### No stimulus–response associations between self, other, left and right

Experiments 4 and 5 tested for any implicit associations between self/left and other/right compared to self/right and other/left. With 1000 ms stimuli in Experiment 4, participants responding with the left hand for self and right for other (mean ± SE RT = 847 ± 57.4 ms, error = 16.7 ± 2.4%) did not perform significantly differently to those using the opposite mapping (RT = 859 ± 73.2 ms, error = 16.4 ± 1.77%, *t*(26) = 0.13, *p* = 0.900). Neither group showed a significant difference between self and other (RT: *t*(13) = 2.1,1.0, *p* = 0.056, 0.336; error: *t*(13) = 0.18, 1.71, *p* = 0.863, 0.336), and these self–other differences also did not differ significantly (i.e., there was no interaction, RT: *t*(26) = 0.99, *p* = 0.331; error: *t*(26) = 0.88, *p* = 0.387). There was also no difference between self-left and self-right groups in Experiment 5’s overall data (mean ± SD RT difference = 89.4 ± 224 ms, *t*(26) = 1.06, *p* = 0.301), or any differences in their self-other differences (i.e., no interaction: RT, *t*(26) = 0.82, *p* = 0.421; error, *t*(26) = 0.44, *p* = 0.665).

### Prior information changes response bias, but does not improve accuracy

**Fig. 4:**
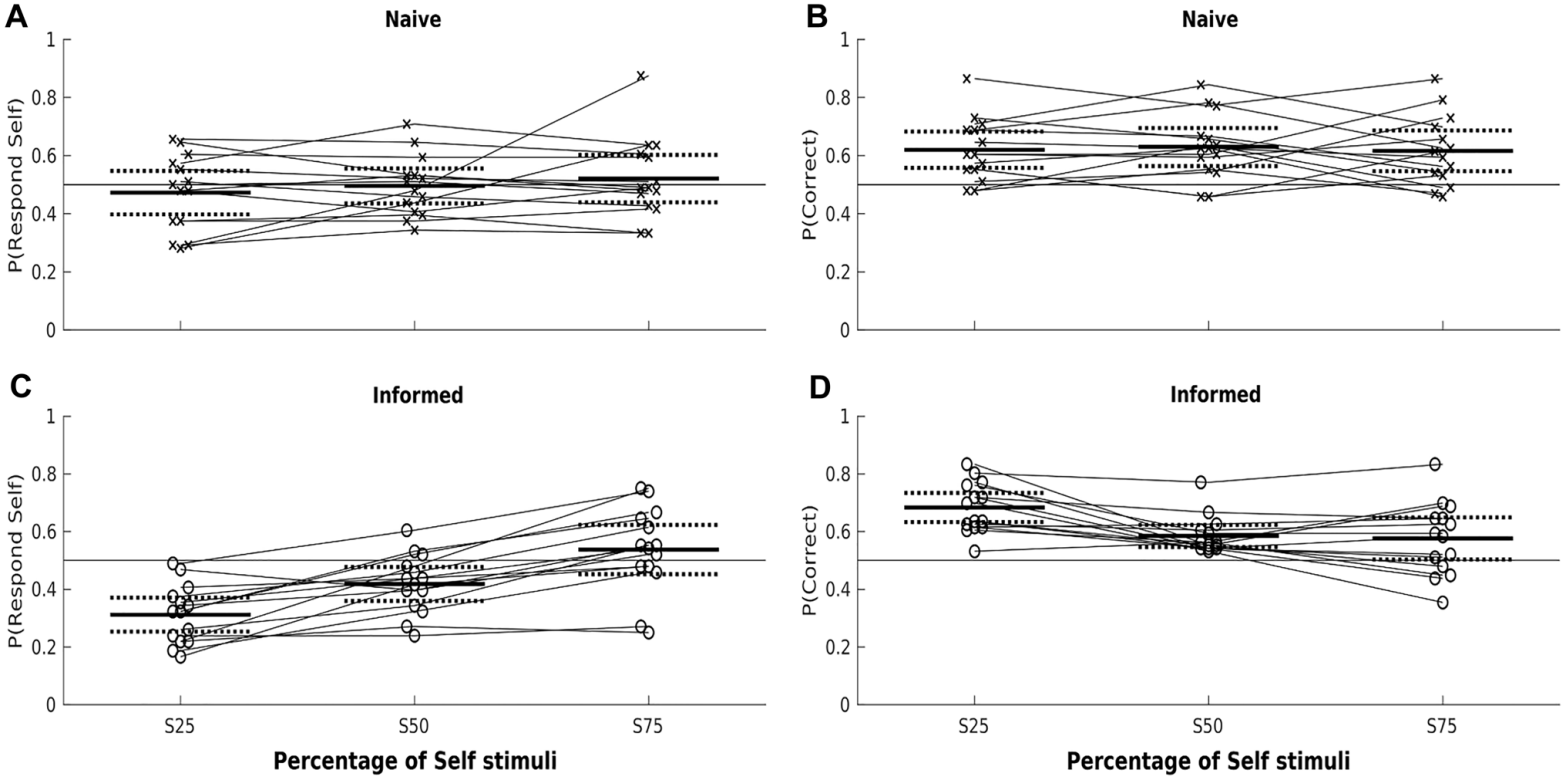
Informing participants about the proportion of self-images changes their self-response frequency but not their overall accuracy. Panels **A** and **C** show the proportion of ‘Self’ responses in a self-other discrimination task; **B** and **D** show the proportion correct responses. The top panels show data for ‘naive’ participants (x) who were told only that the three conditions were different. Bottom panels show participants (o) informed about the percentage of self-stimuli in each block. Within each panel, the three conditions show the percentage of self-stimuli per block (e.g., S25: 25% self, 75% other). Thin lines connect data from the same participant. The data show that naive participants performed similarly across the three blocks, while informed participants adjusted their responses, particularly in the S25 condition. Solid horizontal lines: Means; broken horizontal lines: 95% confidence intervals (showing the two-tailed *t* test comparison with zero)

In Experiment 5, the three blocks of trials had different percentages of self-stimuli (25, 50, 75%), and only half of the participants were informed about this. The experimental manipulations were successful: a significant effect on the percentage of self-responses (means ± SEs = 39.2 ± 2.7, 45.7 ± 2.1, 52.9 ± 2.7%; *F*(2, 52) = 16.3, *p* < 0.001), and a significant interaction with prior information (*F*(2, 52) = 6.82, *p* = 0.002, Fig. 4a, b), but no significant main effect of information, *F*(1,26) = 3.89, *p* = 0.056. The informed group responded ‘self’ slightly less often (M ± SD = 42.3 ± 2.7%) than the naive group (49.6 ± 2.6%).

Similarly, the percentage correct was affected by the percentage of self-stimuli (mean ± SE = 61.5 ± 1.9%, 60.7 ± 1.8%, 59.6 ± 2.3%, *F*(2, 52) = 3.55, *p* = 0.036), and this interacted significantly with being informed (*F*(2, 52) = 3.85, *p* = 0.028, Fig. 4c, d), but there was no main effect of being informed (naive M ± SD = 62.2 ± 9.4%; informed = 61.5 ± 6.8%; *F*(1, 26) = 0.054, *p* = 0.818). Overall, informing participants about the percentage of self-stimuli strongly affected how they responded, but did not improve performance.

### Meta-analysis provides little support for self-advantage effects

**Fig. 5:**
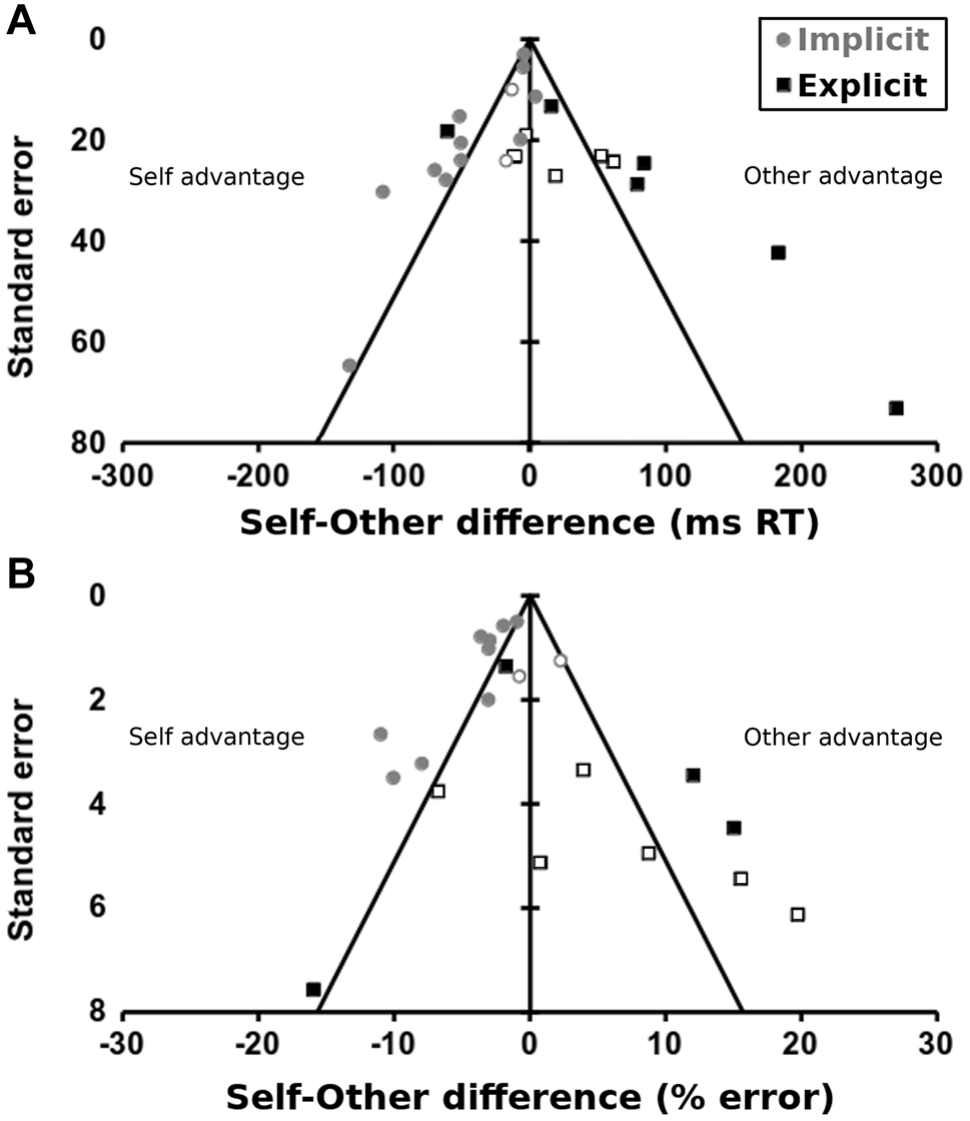
Meta-analysis and funnel-plot of the ‘Self-advantage’ effect shows no overall self-advantage effect and indicates some publication bias. Systematic review identified 20 studies of hand recognition involving static images of participants’ hands. Studies were classified according to whether the recognition task was ‘implicit’ (e.g., same vs. different or left vs. right discrimination, grey circles) or ‘explicit’ (i.e., self vs. other discrimination, black squares). Open symbols: data from the current study. Studies reporting sufficient details to calculate an effect size (*x*-axes: ms reaction time **A**; % error **B**) and its standard error (*y*-axes) for a self-other comparison are included. The data suggest no overall self-advantage effect, but very different distributions of implicit and explicit effect-sizes. Negative values indicate a self-advantage effect, positive a self-disadvantage or other-advantage. The solid black triangle shows the approximate *p* = 0.05 limit—data points inside the triangle show no significant difference between self and other, data points outside show a significant difference at *p* < 0.05

Across all available data, pooling implicit and explicit tasks (Fig. 5), there was no significant self-advantage (or disadvantage) for RTs (M ± SE = − 1.2 ± 12.5, 95% CI = {− 25.6, 23.2}ms, *Z* = − 0.10, *p* = 0.923) or errors (− 0.13 ± 1.68, {− 3.42, 3.15}%, *Z* = − 0.08, *p* = 0.937). There was no apparent publication bias, based on the funnel regression statistic, for RT (*Z* = 1.64, *p* = 0.102) or error data (*Z* = 1.68, *p* = 0.093). The funnel plots appear symmetrical.

The same conclusions were reached for error data from explicit tasks considered alone, but explicit RTs showed a weak self-disadvantage (43.8 ± 20.6 ms, {3.33, 84.2}ms, Z = 2.12, p = 0.034), and evidence of significant bias towards publication of these self-disadvantage effects (Z = 4.37, p < 0.001).

The clearest results were from implicit tasks alone. Both RT (− 31.2 ± 9.04, {− 48.9, − 13.5}ms, *Z* = − 3.45, *p* < 0.001) and error data (− 3.10 ± 0.95, {− 4.97, − 1.24}%, *Z* = − 3.26, *p* = 0.001) showed significant self-advantage effects. But, the available RT (*Z* = 4.65, *p* < 0.001) and error (*Z* = 2.72, *p* = 0.006) data both suggested significant biases for self-advantage effects to be published. Trim-and-fill analysis did not change the error data, but substantially reduced the RT effect after imputing 4 ‘missing’ studies (− 18.9 ± 10.5, {− 39.5, 1.7}ms, *Z* = 1.80, *p* = 0.073).

Very similar conclusions were reached when the current data were removed from the meta-analysis (Supplementary Materials). Overall, there is some evidence of a self-advantage effect for implicit tasks only, but this is complicated by an apparent bias for those effects to be published.

## Discussion

In three groups of participants, and across five experiments, we found no significant self-advantage, but several significant self-disadvantages during explicit recognition of hand photographs. We also found strong biases to respond ‘other’ when the explicit task was more difficult, and no significant improvement for self-left and other-right responses compared to opposite mappings. Informing participants about the likelihood of self-images significantly decreased response biases, but did not improve accuracy. Across the literature, there was no evidence for a general self-advantage, and some evidence for a self-advantage in implicit discrimination (e.g., same/other or left/right), but this was complicated by an apparent publication bias, based on funnel plot asymmetry and the funnel regression statistics.

Before addressing what we consider the main discussion points, some limitations of the study. First, hand photograph recognition tasks like the one that we and the 20 other reviewed studies have used are not assessing ‘self-recognition’ directly. Rather, the experimenters contrived a situation in which computerised and edited images of participants hands are presented on screen. During stimulus generation and presentation, the colours, sizes, and some salient features of the participants’ hands were manipulated or edited out. Furthermore, each participant’s hand was matched with two others. The participants’ ability to distinguish self from other hands in these tasks depends on these editing and matching processes. Removing all identifying features and matching the hands perfectly would make the task impossible. Self-recognition under these conditions is therefore quite arbitrary. The main conclusions from this work should, therefore, be about how participants tend to respond in these tasks; which factors change hand recognition performance; and how participants deal with uncertainty. Whether and how these processes relate to everyday self-recognition, or the self-recognition deficits that can occur following brain injury (e.g., Rossetti et al. 2010; Vallar and Ronchi 2009), are important questions for future work.

### Weak evidence for a self-advantage effect

The failure to find a significant self-advantage effect, at least in the left-versus-right task (Experiment 1), is unexpected, given the weight of prior evidence for implicit self-advantages (16/17 RT and 15/17 error effects) and explicit self-disadvantages (7/8 RT, but only 4/9 error effects). Across the previous implicit tasks, the mean self-advantage was 35 ms, and the mean effect size, Cohen’s *d*, was 0.443. To achieve 80% statistical power, studies would need at least 40 participants, but none, including the present five, had more than 30. Studies in this field are underpowered to detect a possible self-advantage. Furthermore, the meta-analytic funnel plot revealed that implicit self-advantages seem to converge towards a peak at zero effect, while the published self-disadvantages show a similar, but opposite trend. It is possible that self-advantage effects are more likely to be published if they arise from implicit tasks, and self-disadvantages from explicit tasks. Meta-analysis is only as reliable as the data itself, however, and 13 relevant studies did not report a standard error, so these effects could not be included. Based on the present study, the self-advantage effect may need to be re-assessed with larger, ideally preregistered, experiments.

### Strong response biases in explicit self-other discrimination

During our explicit self-versus-other tasks, participants did not respond to ‘self’ and ‘other’ stimuli equally. Instead of making a self versus other discrimination, participants seemed to perform self-detection instead, responding ‘self’ more when the task was easier, perhaps when they were more confident that the hand was theirs. When task difficulty increased, participants’ criterion to respond ‘self’ became more conservative, resulting in many more errors for self than other stimuli. These strong biases were reduced slightly by instructing participants about the proportion of self-stimuli.

Moving from implicit to explicit self-recognition changed the way in which participants respond. When shown an image of a hand and asked whose it is, the default assumption appears to be that it is not theirs, and good evidence is required for them to respond ‘self’. The literature on self-recognition contains many different tasks. We focussed only on recognition of single hand photographs, but other self-recognition tasks have used video (e.g., Daprati et al. 1997), computer-generated (e.g., Franck et al. 2001), or symbolic (e.g., Knoblich and Prinz 2001) representations of participants’ hands and their movements. Despite the apparent similarity, self-recognition in these circumstances involves different task requirements. In some early self-recognition tasks (e.g., Daprati et al. 1997; van den Bos and Jeannerod 2002), participants had to say whether a video representation of their hand and/or its movement on a screen was theirs (or caused by them) or someone else’s. The participants were aware that their own hand movements were being recorded or tracked, that sometimes it would not be their hand, and the hands were often disguised with gloves to make self and other as difficult as possible to distinguish. In some studies, the participants were deceived—there was no ‘other’ hand at all (e.g., Farrer et al. 2008). In these situations, participants tended to show a bias to respond ‘self’ when the task was difficult—opposite to the biases we found for hand image recognition. There seem to be different default responses for recognition of self-images and of self-movement, which may depend on how the task is framed.

Video-based task instructions have sometimes been framed, for example, as: “*Did the movement you saw on the screen exactly correspond to that you have made with your hand?*” (Franck et al. 2001), to which the correct answer is always “no”—a two-dimensional, delayed, shifted or rotated computer representation can never exactly correspond to your own hand. Instead of correctly saying “no” on every trial (such annoying participants would be excluded as, for example, by Arikan et al. 2019!), participants play along and seem to treat the task as a ‘difference detection’ task—at what level of stimulus manipulation can they detect a difference between what they did or felt, and what they saw on the screen (or, perhaps, between the lowest level of stimulus manipulation that they can use as an implicit or internal standard against which to compare other stimuli). If they detect no difference, they should say ‘self’. Experimental results in self-recognition tasks are strongly affected by participants’ response criteria when the task is difficult, but in opposite directions for images and videos: the default response is ‘other’ for briefly-presented photographs, but ‘self’ for videos of hands disguised by gloves.

To avoid these forms of response bias, and focus on self-recognition ability (i.e., detection or discrimination threshold), participants should be fully informed and presented on every trial with two stimuli, either sequentially or simultaneously. One stimulus should always be ‘self’, one always ‘other’, and the participant must choose which is theirs (or, for balance, which is not theirs). Under such an experimental design, guessing and strong response-biases would lead to performance not significantly different from 50%, and the participant could then be excluded for task failure. The self-stimulus could then be delayed, shifted, morphed, rotated, or undergo any other experimental manipulation, to examine its effects on self-recognition ability. As long as there is always a correct answer—always one stimulus that is more ‘self’ or less distorted than the other—then this two-interval or two-alternative forced-choice experimental design can tell us more about self-recognition ability and less about response bias. A series of such experiments has been conducted by Hoover and Harris (2012) and Hoover et al. 2016), and these provide an excellent basis for further work questioning whether self is indeed special (Gillihan and Farah 2005).

## Supplementary Information

Below is the link to the electronic supplementary material.

Supplementary file1 (ODS 293 kb)

Supplementary file2 (DOC 984 kb)

## Data Availability

All stimuli, raw data, analytic code and the meta-analysis are freely available for re-use at https://osf.io/4ydzh/.
