# Supplementary material for: No self-advantage in recognizing photographs of one’s own hand: experimental and meta-analytic evidence
Source: Exp Brain Res. 2022 May 20;240(9):2221–33. doi: 10.1007/s00221-022-06385-9 (PMC9458563; doi:10.1007/s00221-022-06385-9)
Supplement: Supplementary file 2 — Supplementary file2 (DOC 984 kb) [file 221_2022_6385_MOESM2_ESM.doc]

**Supplementary Figures**


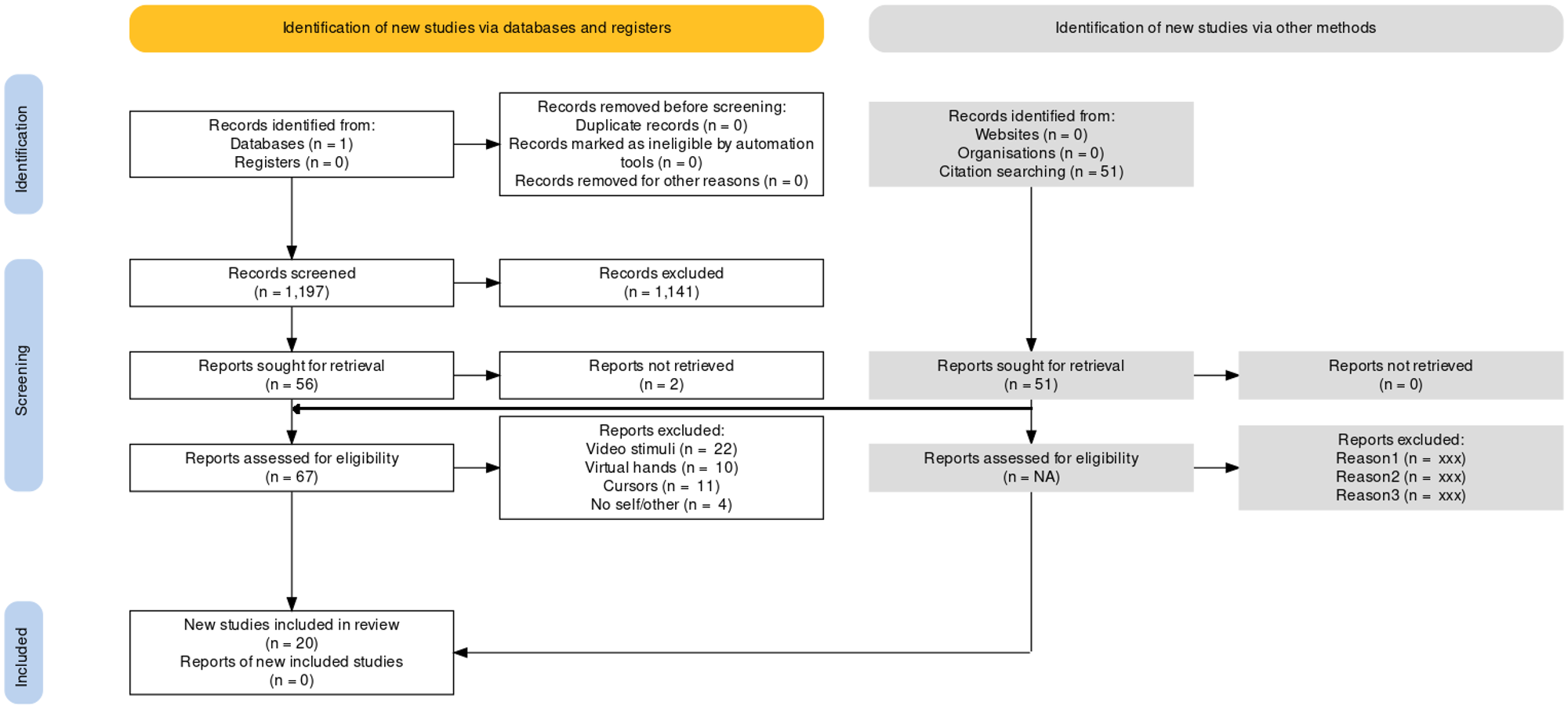
Supplementary Figure 1. PRISMA flowchart. Created at <https://estech.shinyapps.io/prisma_flowdiagram/> and slightly modified.


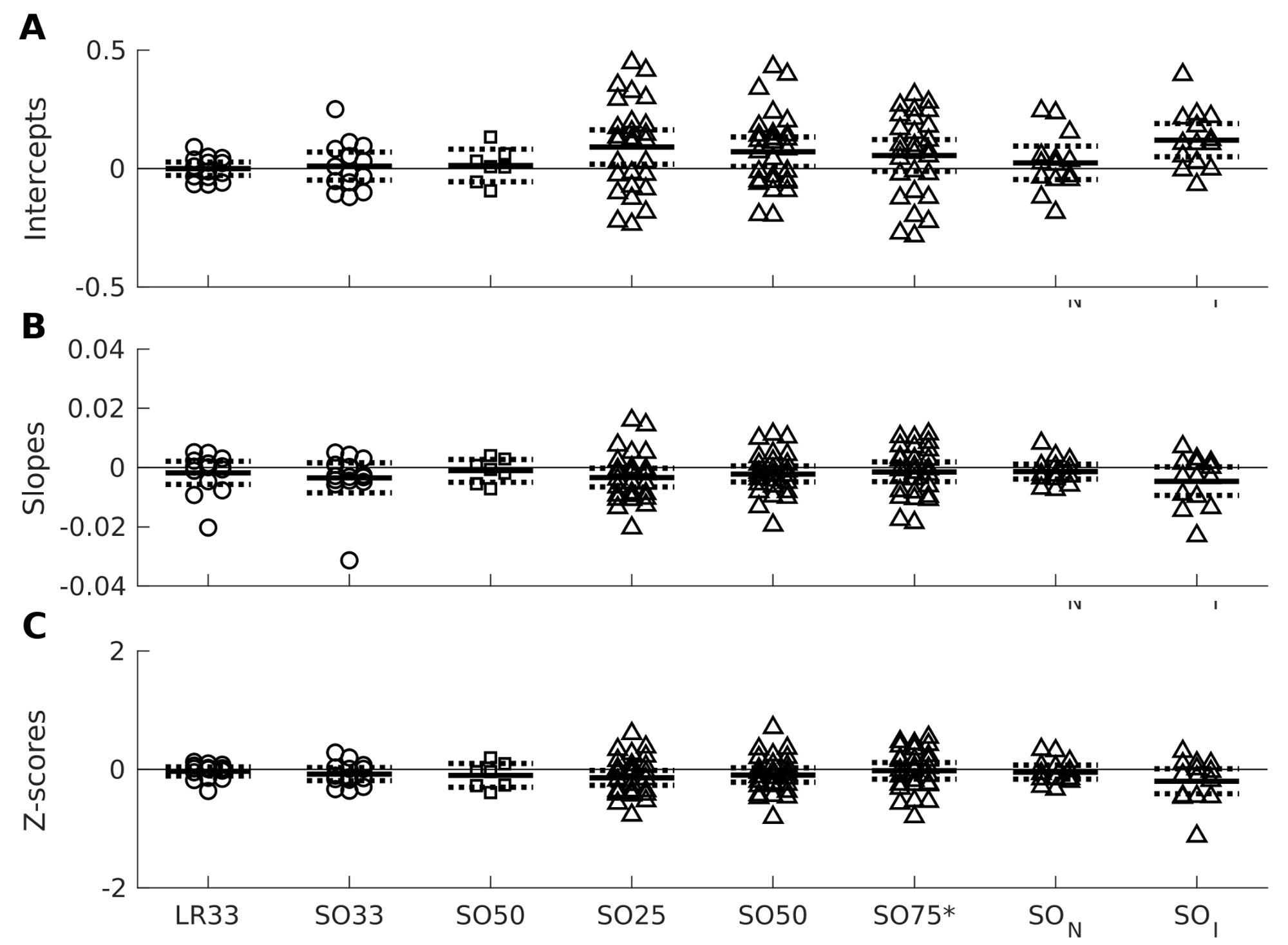
**Supplementary Figure 2. Task difficulty only weakly affects the relative speed of participants’ responses to self and other stimuli**. For each task with stimuli presented for varying durations, log10(RT) and error data were fitted with linear and logistic functions respectively, separately for self and other stimuli across experimental conditions (E1b to E5:I). Differences in fit parameters (Self-Other) for reaction times are shown in the figure. There were some significant differences between self and other fit parameters (Table 3), but these were not as strong or consistent as for the percentage error data, and will not be interpreted further. Stimulus duration was on the x-axis. Each fit gave an intercept (**A**), slope (**B**) and r-value. R-values were transformed to Z-scores (**C**) to allow parametric analysis. N: Naive participants only; I: Informed participants only. Solid horizontal lines: Means; broken horizontal lines: 95% confidence intervals (showing the two-tailed t-test comparison with zero). Circles: Group 1; Squares: Group 2; Triangles: Group 3. *One or two extreme-outlying statistics were removed from each of these analyses.


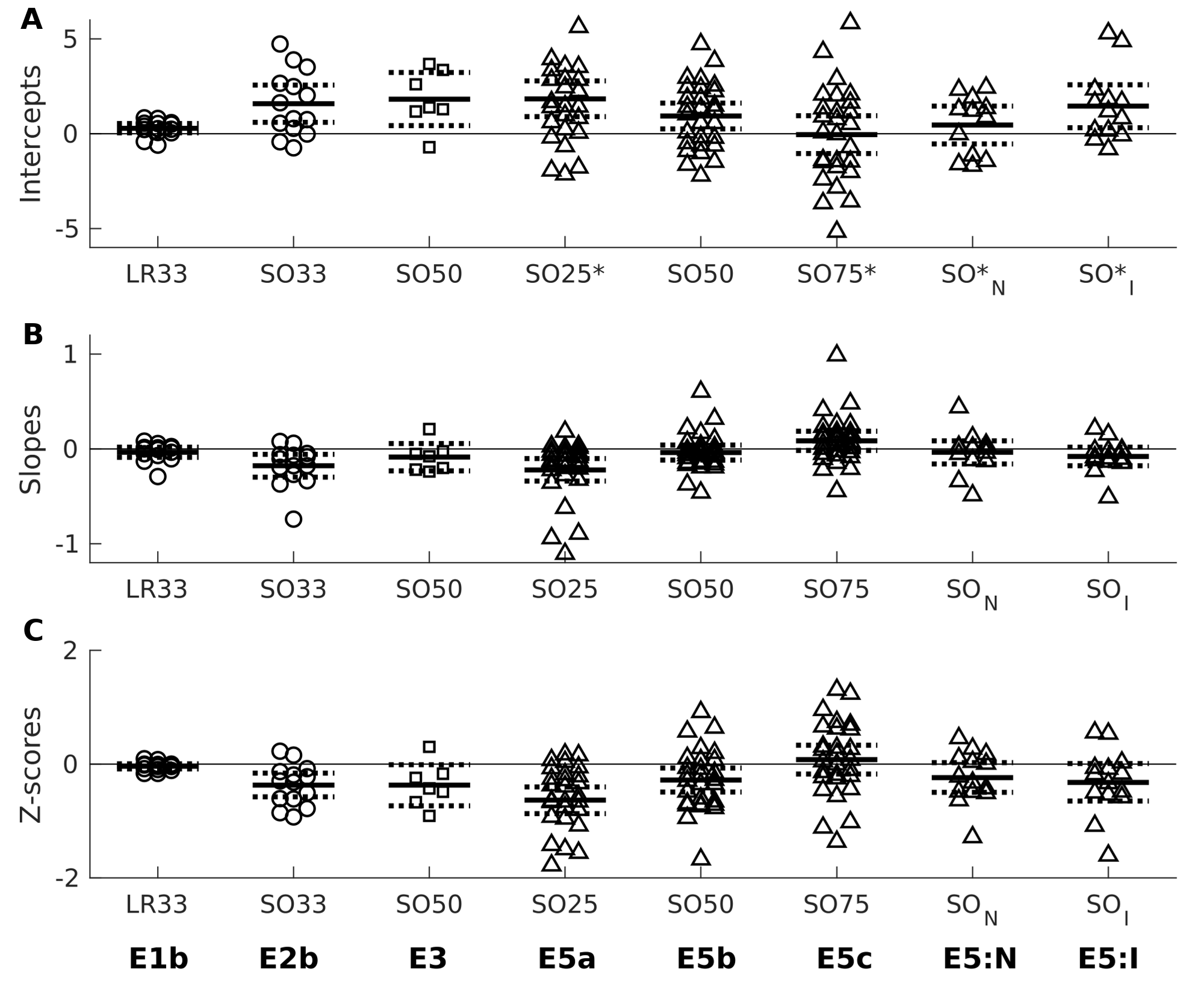


**Supplementary Figure 3. Task difficulty affects how participants respond to self and other stimuli**. For each task with stimuli presented for varying durations, log10(RT) and error data were fit with linear and logistic functions respectively, separately for self and other stimuli across experimental conditions (E1b to E5:I). Differences in fit parameters (Self-Other) for percentage errors are shown in the figure (RT data are in Supplementary Figure 1). The intercepts (**A**) were higher for Self than Other stimuli in all tasks except in Experiment 5 when 75% of stimuli were self or the participants were not informed about the proportion of self stimuli. Data for slopes (**B**) and Z-scores (**C**) showed similar trends, but less strongly. Stimulus duration was on the x-axis. Each fit gave an intercept, slope and r-value. R-values were transformed to Z-scores to allow parametric analysis. N: Naive participants only; I: Informed participants only. Solid horizontal lines: Means; broken horizontal lines: 95% confidence intervals (showing the two-tailed t-test comparison with zero). Circles: Group 1; Squares: Group 2; Triangles: Group 3. * One or two extreme-outlying statistics were removed from each of these analyses. Several significant differences for RTs (Table 3) and errors (Table 4) showed that self stimuli were associated with higher intercepts (longer RT, more errors), more negative slopes (i.e., a greater effect of stimulus duration), and stronger or more negative Z-scores (a greater standardised effect of stimulus duration) than other stimuli.


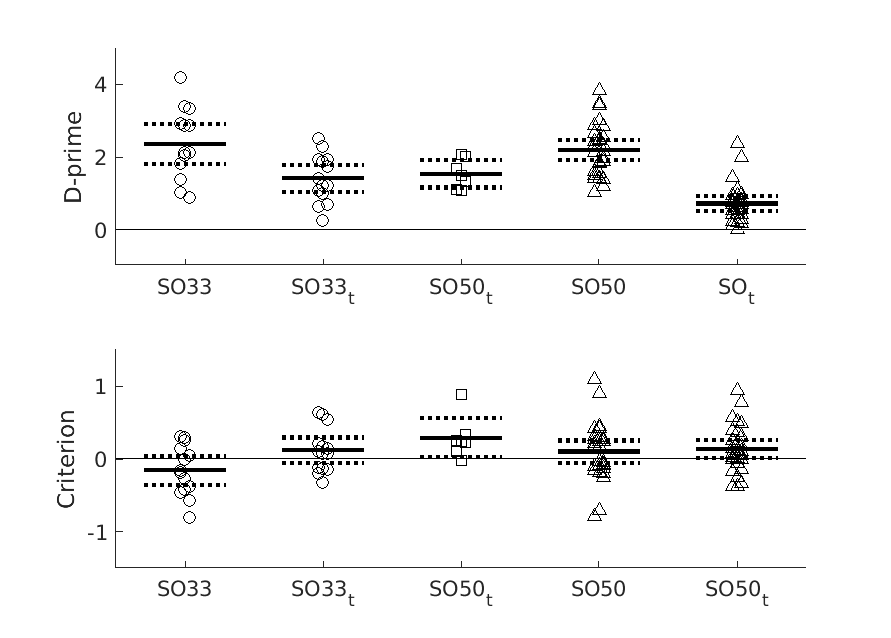


**Supplementary Figure 4: Across all experimental conditions, participants perform the task well (d-prime mostly>1) and do not show strong response biases (criterion ~0).** For the purpose of these signal detection analyses, ‘self’ stimuli were defined as the ‘signal’ and ‘other’ stimuli as ‘noise’. D-prime was calcualted as the difference in Z-transformed proportions between Hits (correct ‘self’ responses to ‘self’ stimuli) and False Alarms (incorrect ‘self’ responses to ‘other’ stimuli). Ceiling (p=1) and floor (p=0) proportions were adjusted by subtracting or adding 1/2N, respectively, where N is the total number of ‘self’ or ‘other’ trials, respectively. d-prime=Z(Hits)-Z(False alarms). Criterion was calculated as C=-0.5*(Z(Hits)+Z(False alarms). Means; broken horizontal lines: 95% confidence intervals (showing the two-tailed t-test comparison with zero). Circles: Group 1; Squares: Group 2; Triangles: Group 3.

**
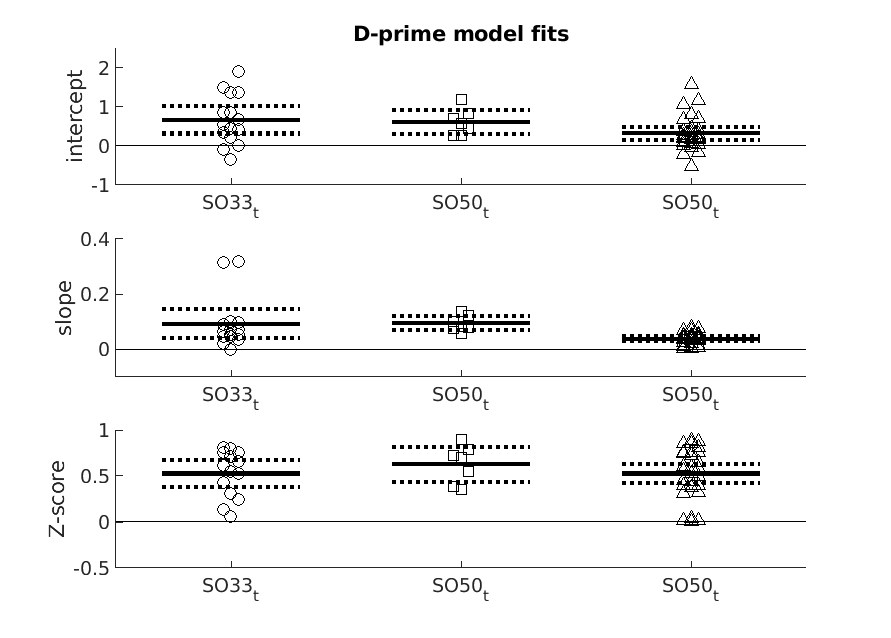
**

**Supplementary Figure 5. D-prime scores increase with longer stimulus duration.** The d-prime scores were fit as a function of stimulus duration using linear regression. The model fit parameters – intercept, slope, and Z-transformed r-coefficients were significantly positive across all groups (i.e., the confidence intervals did not include zero). Means; broken horizontal lines: 95% confidence intervals (showing the two-tailed t-test comparison with zero). Circles: Group 1; Squares: Group 2; Triangles: Group 3.

**
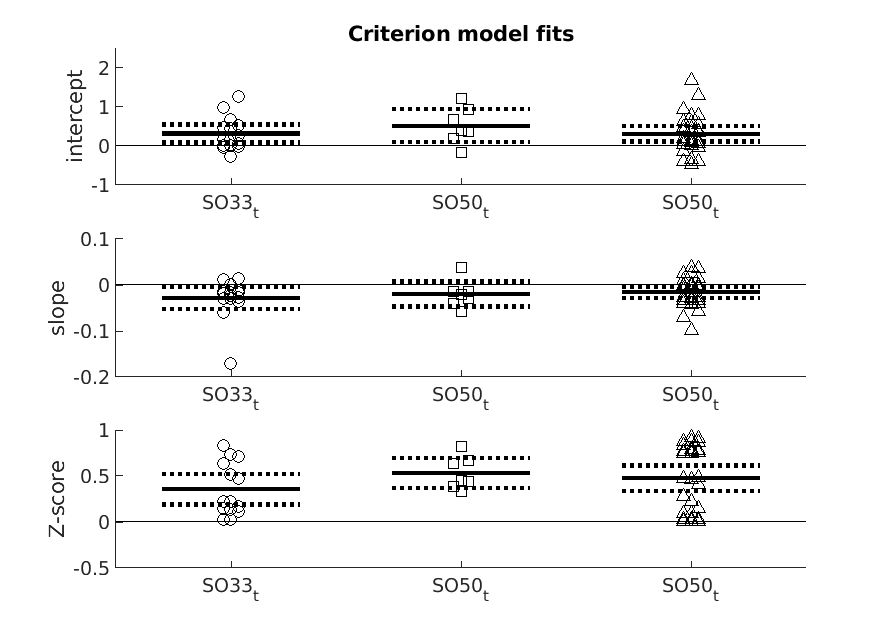
**

**Supplementary Figure 6. Criterion decreases with longer stimulus duration.** The criterion scores were fit as a function of stimulus duration using linear regression. The model fit parameters – intercept, slope, and Z-transformed r-coefficients were significantly negative in all groups (i.e., the confidence intervals did not include zero), apart from the slopes in Experiment 3, t(6)=-1.76, p=.128. Means; broken horizontal lines: 95% confidence intervals (showing the two-tailed t-test comparison with zero). Circles: Group 1; Squares: Group 2; Triangles: Group 3.

**Table S1 Signal detection analyses for Self vs. Other tasks**

| **E** | **P(Self)** | **Dur.** | **N** | **d-prime** | | | **Criterion** | | |
| --- | --- | --- | --- | --- | --- | --- | --- | --- | --- |
|  |  | **ms** |  | **Mean**  **(SE)** | **t** | **p** | **Mean**  **(SE)** | **t** | **p** |
| 2a | 0.33 | 1000 | 14 | 2.36  (0.255) | 9.23 | <.001 | -0.156  (0.093) | -1.68 | 0.12 |
| 2b |  | 17-  533 |  | 1.41  (0.175) | 8.04 | <.001 | 0.12  (0.081) | 1.48 | 0.16 |
| 3 | 0.5 | 17-  533 | 7 | 1.54  (0.154) | 9.99 | <.001 | 0.291  (0.11) | 2.66 | 0.04 |
| 4 | 0.5 | 1000 | 28 | 2.19  (0.135) | 16.2 | <.001 | 0.101  (0.0745) | 1.36 | 0.19 |
| 5 | 0.75 | 17-  533 |  | 0.718  (0.0992) | 7.24 | <.001 | 0.137  (0.0618) | 2.22 | 0.03 |

*E: Experiment and condition; P(Self): Proportion of self stimuli. Dur.: Stimulus duration; t: t-statistic for Self-Other Difference; Negative criterion=bias to respond ‘self’; p: p-value comparing each parameter with zero. For d-prime, significant effects mean that participants could perform the self vs. other task better than chance. For criterion, significant effects mean that participants’ responses were biased.*

**Table S2. Signal detection analyses for Self vs. Other tasks: regression of SDT parameters against stimulus duration**

| **E** | **d-prime** | | | | | | | | | **Criterion** | | | | | | | | |
| --- | --- | --- | --- | --- | --- | --- | --- | --- | --- | --- | --- | --- | --- | --- | --- | --- | --- | --- |
|  | **Intercept** | | | **Slope** | | | **Z-score** | | | **Intercept** | | | **Slope** | | | **Z-score** | | |
|  | **M**  **(SE)** | **t** | **p** | **M*1000**  **(SE)** | **t** | **p** | **M**  **(SE)** | **t** | **p** | **M**  **(SE)** | **t** | **p** | **M*1000**  **(SE)** | **t** | **p** | **M**  **(SE)** | **t** | **p** |
| 2b | 0.669  (0.165) | 4.05 | .001 | 0.928  (0.245) | 3.78 | .002 | 0.964  (0.106) | 9.07 | <.001 | 0.313  (0.108) | 2.91 | .011 | -0.282  (0.112) | -2.52 | .025 | -0.589  (0.159) | -3.7 | .003 |
| 3 | 0.607  (0.125) | 4.87 | .003 | 0.944  (0.104) | 9.05 | <.001 | 1.15  (0.148) | 7.76 | <.001 | 0.516  (0.174) | 2.97 | .025 | -0.196  (0.111) | -1.76 | .128 | -0.73  (0.279) | -2.61 | .04 |
| 5 | 0.317  (0.083) | 3.83 | <.001 | 0.382  (0.04) | 9.44 | <.001 | 0.972  (0.090) | 10.8 | <.001 | 0.302  (0.094) | 3.21 | .003 | -0.157  (0.06) | -2.64 | .014 | -0.453  (0.196) | -2.31 | .029 |

*E: Experiment and condition; M: Mean; SE: Standard error; t: t-statistic comparing each parameter with zero; p: uncorrected p-value. Significant effects show that stimulus duration had an effect on the parameter, increasing D-prime and decreasing criterion as stimulus duration increased.*


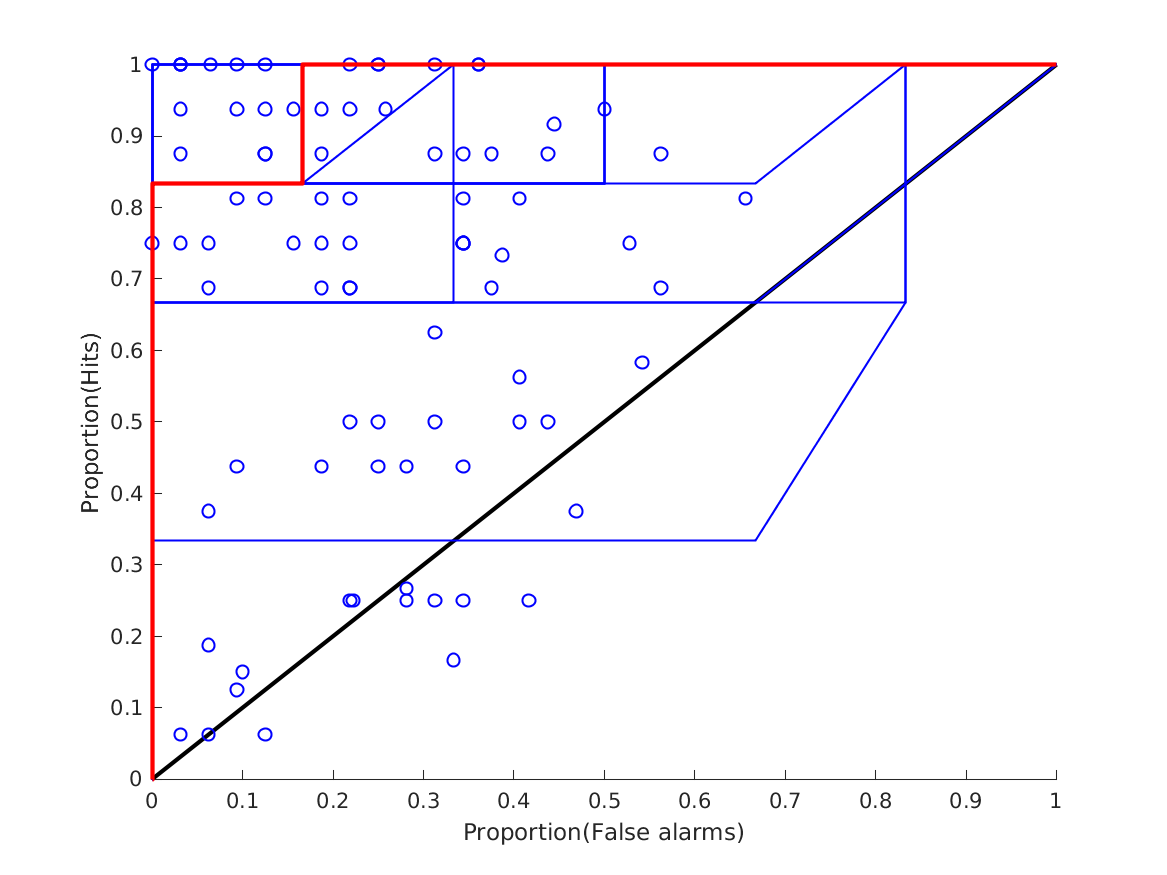
*Supplementary Figure 7: Receiver-operating characteristic curves for Experiment 2b. For each participant, the proportion of false alarms (x-axis) for each of 6 durations was plot against the proportion of hits (y-axis) – (6 blue circles per participant). MATLAB’s roc function was used to create the ROC curve for each participant (blue lines). The red line is the ROC curve using the mean proportions across all participants. The black diagonal line represents chance performance where d-prime=0. Most datapoints and most curves are above the diagonal, showing that the task was being performed correctly. Overall performance at discriminating self from other was good.*


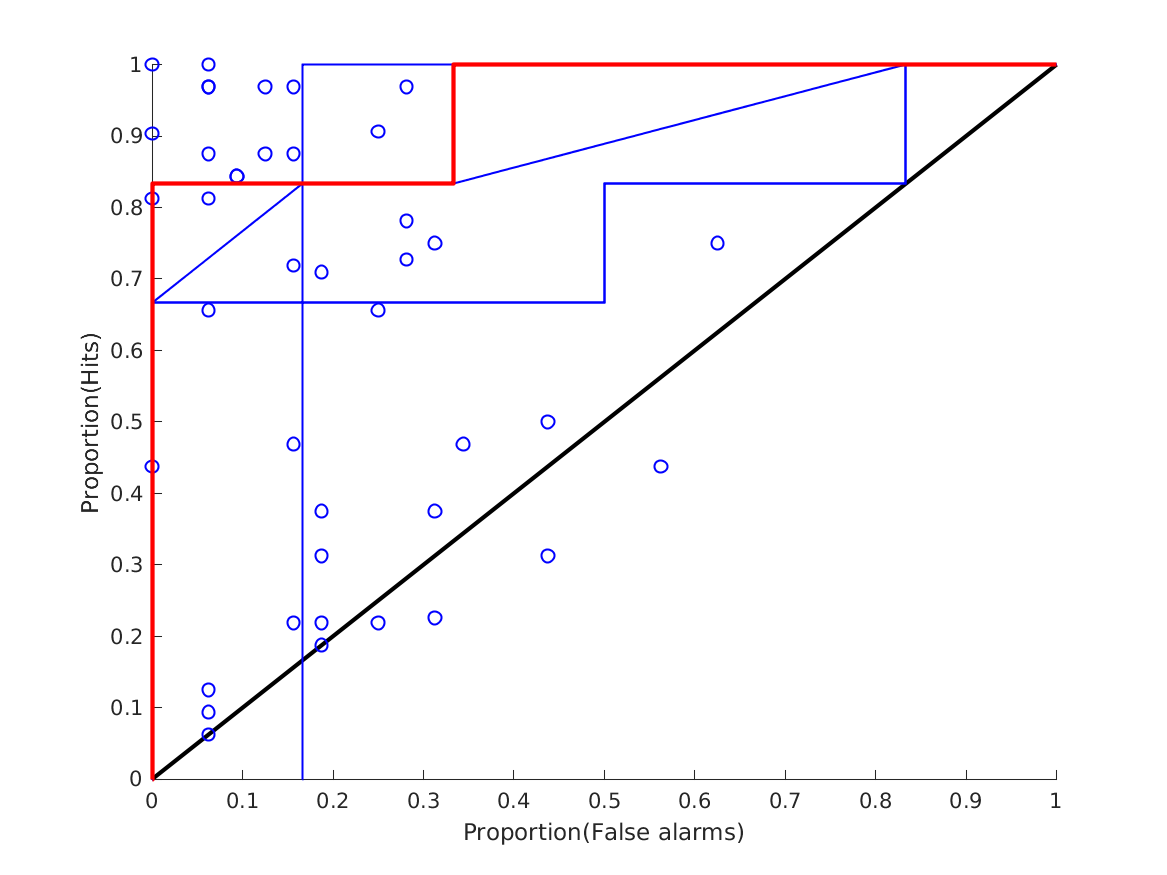
*Supplementary Figure 8: Receiver-operating characteristic curves for Experiment 3. For each participant, the proportion of false alarms (x-axis) for each of 6 durations was plot against the proportion of hits (y-axis) – (6 blue circles per participant). MATLAB’s roc function was used to create the ROC curve for each participant (blue lines). The red line is the ROC curve using the mean proportions across all participants. The black diagonal line represents chance performance where d-prime=0. Most datapoints and most curves are above the diagonal, showing that the task was being performed correctly. Overall performance at discriminating self from other was good.*


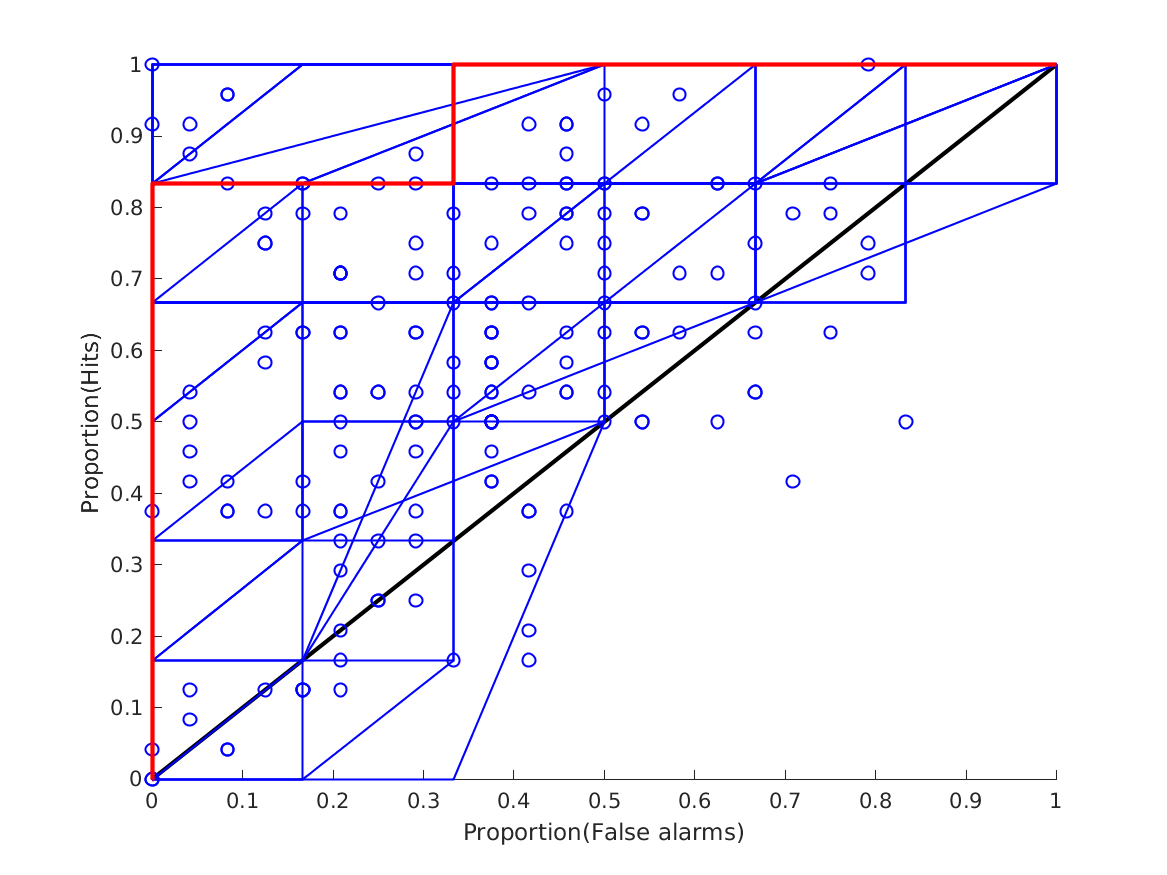
*Supplementary Figure 9: Receiver-operating characteristic curves for Experiment 5. For each participant, the proportion of false alarms (x-axis) for each of 6 durations was plot against the proportion of hits (y-axis) – (6 blue circles per participant). MATLAB’s roc function was used to create the ROC curve for each participant (blue lines). The red line is the ROC curve using the mean proportions across all participants. The black diagonal line represents chance performance where d-prime=0. Most datapoints and most curves are above the diagonal, showing that the task was being performed correctly. Overall performance at discriminating self from other was good.*
